# Luminescence of Binary-Doped Silica Aerogel Powders: A Two-Step Sol-Gel Approach

**DOI:** 10.3390/gels10020104

**Published:** 2024-01-27

**Authors:** Dimitar Shandurkov, Nina Danchova, Tony Spassov, Vesselin Petrov, Stoyan Gutzov

**Affiliations:** Faculty of Chemistry and Pharmacy, Sofia University St. Kliment Ohridski, 1164 Sofia, Bulgaria; fhds@chem.uni-sofia.bg (D.S.); ndanchova@chem.uni-sofia.bg (N.D.); tspassov@chem.uni-sofia.bg (T.S.);

**Keywords:** aerogel powders, luminescence, europium, terbium, energy transfer, thermal conductivity

## Abstract

In this study, we report a novel synthesis of hydrophobic silica aerogel powder composites, functionalized and binary-doped with [Tb(phen)_2_](NO_3_)_3_ and [Eu(phen)_2_](NO_3_)_3_ nanocrystals, employing a two-step sol-gel methodology. The investigation delves into the structural elucidation, optical properties and thermal conductivity of these functionalized Tb(III)-Eu(III) composites. Our analysis includes diffuse reflectance spectra and excitation and luminescence spectra, highlighting the quantum yields of composites with varying chemical compositions. Remarkably, these samples exhibit a strong luminescence, with distinct hues of red or green based on the specific doping type and level. The detailed examination of excitation spectra and quantum yields establishes robust energy-transfer mechanisms from the 1,10-phenanthroline molecule to the lanthanide ions. Notably, our study uncovers a Tb^3^⁺→Eu^3^⁺ energy-transfer phenomenon within the binary functionalized samples, providing compelling evidence for a structural formation process occurring within the mesoporous framework of the aerogel powders.

## 1. Introduction

Optical composite materials, based on aerogels and doped with functional nanocrystallites, are increasingly important in LED technologies, microelectronics and sensorics. Due to their high specific surface area and porosity, silica aerogel matrices have the possibility of incorporating hybrid molecules and d- and f-ions with desired electrical and optical properties [1]. This is the reason for an increasing number of papers dealing with the preparation and characterization of functional aerogel composites and aerogels including their physical properties and physicochemical preparation strategies.

The first known publication that we encountered with optical composites based on silica hydrophobic aerogels shows the possibility of obtaining composites with the composition SiO_2_(aerogel):[Eu(phen)_2_](NO_3_)_3_ using a two-step sol-gel process [2].

In the next contribution, knowledge of this process was extended by finding dependences of the quantum yield (QY) and spectral intensities of SiO_2_:[Eu(phen)_2_](NO_3_)_3_ and SiO_2_:[Tb(phen)_2_](NO_3_)_3_ composites on the degree of hydrophobicity, α, of the starting aerogel matrix [3]. In the same paper, an increased thermal stability of these composites, up to 200 °C, is shown, compared to that of hydrophilic sol-gel composites. Thus, the hydrophobization of silicate surfaces affects the quantum yield of the obtained luminescent materials, reducing the concentration of surface OH groups and at the same time increasing the thermal stability of the obtained aerogel granules [4]. Stable green- or red-emitting materials enable the production of LED additives and the production of multi-color-emitting aerogel powders [5].

The aim of the present contribution is to obtain SiO_2_(aerogel):[Eu(phen)_2_](NO_3_)_3_ and [Tb(phen)_2_](NO_3_)_3_ binary optical composites using a two-step sol-gel approach and to investigate their optical properties and thermal conductivity as a part of the following framework: the preparation–structure–properties of gel composites. To the best of our knowledge, there are no contributions dealing with binary hybrid nanocrystallites incorporated into silica aerogels. The spectral properties of the activator compounds [Tb(phen)_2_](NO_3_)_3_ and [Eu(phen)_2_](NO_3_)_3_ could be an indicator for phase formation, which takes place in the porous network of the aerogel matrixes.

Inorganic silicate materials containing Eu^3+^ and Tb^3+^ are already known from a number of publications with a theoretical and applied focus [6,7,8,9,10,11]. Moreover, binary lanthanide aerogels are prepared during the search for porous materials with suitable optical and catalytic activities [12]. The mixed binary and ternary lanthanide oxide phases are also well-known examples of high-entropy materials [13].

## 2. Experimental Results

**Figure 1:**
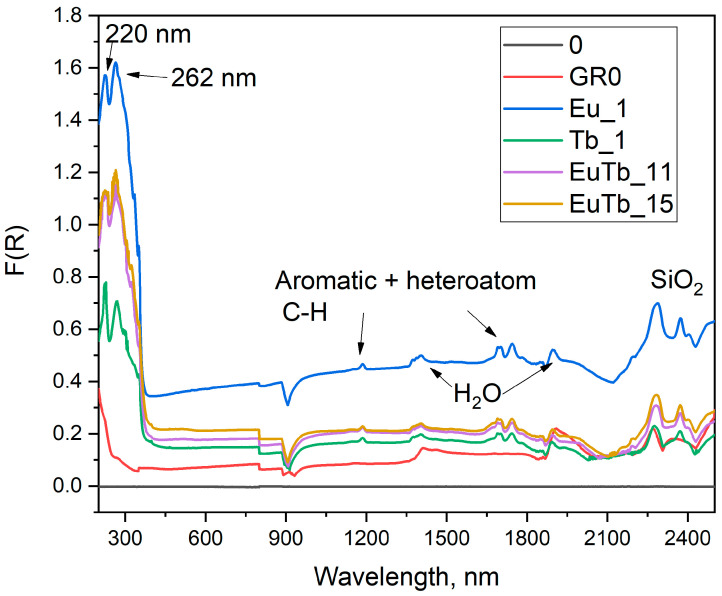
UV-Vis-NIR spectra of the samples. Notations: 0—reference; GR0—non-doped SiO_2_ sol-gel sample. The chemistry of the samples is given in Table 1 and Table 2. Eu_Tb denotes binary-doped composites; Eu_1 and Tb_1 contain Eu^3+^ or Tb^3+^ only.

The UV-Vis-NIR spectra of binary-doped samples and non-hydrophobized and non-functionalized silica powders (notes GR0) are shown in Figure 1. All silica samples display overtones and combinational vibrations attributed to SiO_2_ in the range of 2250–2400 nm. Even the hydrophobic samples have some traces of residual water according to the spectra in the NIR region. Water molecules have two intense overtones in the near-infrared region. The peaks are centered around 1940 and 1450 nm. The luminescent samples also display peaks at 1186 nm and doublets at 1696 and 1745 nm. These bands are associated with combinational and overtone vibrations of C-H groups in aromatic compounds; however, the positions of the maxima are slightly shifted compared to C-H energies in pure benzene. This means they most likely originate from the 1,10-phenanthroline (phen) molecule, and the shift of the maxima is due to the presence of an N-heteroatom in the aromatic ring of the molecule [14].

**Figure 2:**
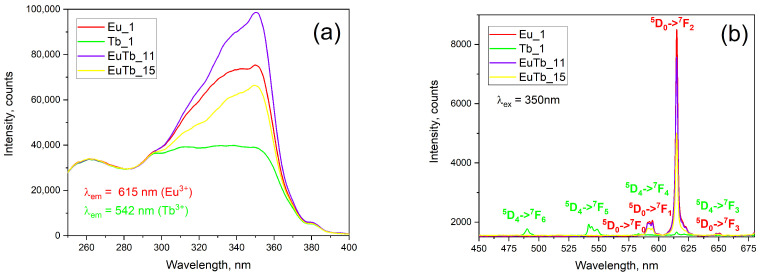
(**a**) Excitation spectra of the luminescent composite sample; (**b**) Emission spectra of the samples. Electronic f-f transitions are given: green for Tb^3+^ and red for Eu^3+^ ions. The chemistry of the samples is given in Table 1 and Table 2. Eu_Tb denotes binary-doped composites; Eu_1 and Tb_1 contain Eu^3+^ or Tb^3+^ only.

Figure 2a,b shows the excitation and emission spectra, respectively, of the composite samples. The excitation spectra do not follow the reflectance spectra.

The thermal properties of the composites are summarized in Table 1. The table contains the degree of hydrophobicity (α), the thermal conductivity (λ), the effusivity (e), and the bulk density (ρ) of the samples.

The aerogel-like character of the functionalized composites investigated here is demonstrated by their low thermal conductivities, which comes from their high porosity. Such an observation proves the effectivity of the aerogel powder drying conditions applied for hydrophilic and hydrophobic species. The thermal properties of low-doped, hydrophobic aerogel powders with the composition SiO_2_:[Ln(phen)_2_](NO_3_)_3_ and n_Ln_/n_SiO2_ = 0.0035 and α = 1.1 [3] are very close to that of highly doped composites: λ = 0.048 W/m.K, e = 76 W.s^1/2^/m^2^.K, and a density of 0.18 g/cm^3^.

The thermal conductivity and effusivity of the pure, powdered dopants—[Tb(phen)_2_](NO_3_)_3_ and [Eu(phen)_2_](NO_3_)_3_—are 0.057 W/m^2^.K and 0.059 W/m.K and 116.5 W.s^½^/m^2^.K and 125.2 W.s^1/2^/m^2^.K, respectively.

**Figure 3:**
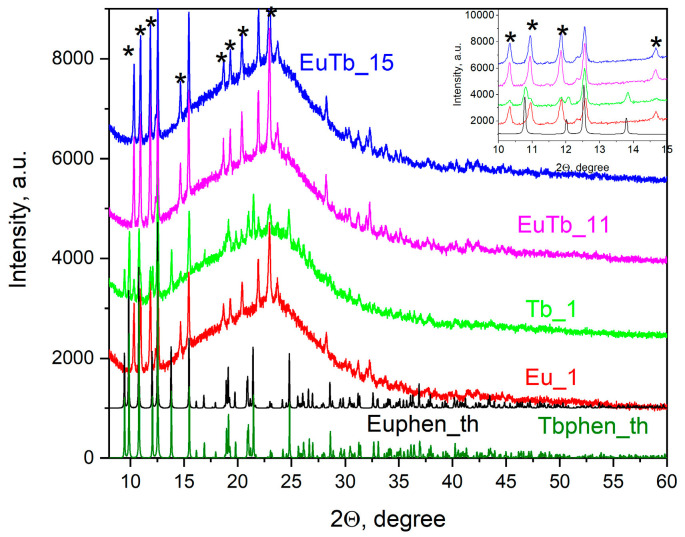
XRD patterns of the binary composite samples. The peaks originating from the second [Eu(phen)_2_](NO_3_)_3_ phase are marked with *. The notations Euphen_th and Tbphen_th refer to theoretical X-ray diffraction patterns of the pure crystalline components: [Eu(phen)_2_](NO_3_)_3_ and [Tb(phen)_2_](NO_3_)_3_.

X-ray diffraction measurements prove that the optically active nanocrystallites in this study display a structural polymorphism (Figure 3). The [Eu(phen)_2_](NO_3_)_3_ and [Tb(phen)_2_](NO_3_)_3_ are isostructural, with small differences in the cell parameters. However, [Eu(phen)_2_](NO_3_)_3_ has two polymorphs, formed depending on the reaction conditions [14]. Lindenberg et al. [14] provide the following cell parameters for the second [Eu(phen)_2_](NO_3_)_3_ phase: a = 9.515, b = 15.454, c = 17.176 Å, and β = 93.45° with a space group of P2_1_/c. The pure powdered [Eu(phen)_2_](NO_3_)_3_ complex crystallizes in a monoclinic phase, as described by Mirochnik et al. [15]. The XRD pattern of this phase is shown in Figure 3 as Euphen_th (black line), while the theoretical patterns of the [Tb(phen)_2_](NO_3_)_3_ are given as Tbphen_th (green line). The PowderCell program [16] was applied to construct the XRD pattern using data collected from the crystal structure information. The crystals are monoclinic, with the space group C2/c. The cell parameters are a = 11.168(1), b = 17.976(2), c = 13.053(1) Å, and β = 100.577(2)°. The XRD pattern for the Tb^3+^ complex was constructed using structure data from the literature [17]. Hence, being isostructural, the two complexes display very similar XRD patterns.

In agreement with the above-mentioned results, the X-ray diffraction patterns here show the presence of both structures, and the peaks of the phase observed by Lindenberg et al. [14] are marked with *. Thus, we can conclude that a mixture of the two polymorphic forms of [Ln(phen)_2_](NO_3_)_3_ is obtained in the pores of the silica gels during the in-situ preparation procedure. The mean crystallite size of the crystallites obtained by the X-ray diffraction analysis is about 20 nm.

No diffraction patterns originating from Eu(NO_3_)_3_, Tb(NO_3_)_3_, or solid 1,10-phenanthroline and their possible crystal hydrates were detected. It should also be stressed that the interpretation of the XRD pattern of the samples is hampered by the low concentration of the dopant (1%) and the presence of an intense, broad amorphous halo originating from the silica matrix [4]. Therefore, the formation of a new nanostructure in the pores of the silica matrix with the composition [Eu,Tb(phen)_2_](NO_3_)_3_ cannot be excluded.

## 3. Discussion

### 3.1. Optical Properties

Due to the low concentration of the Ln^3+^ (1%) complex in the matrix, the f-f transition in the ions is not visible in the reflection spectra of the samples (Figure 1). These transitions are forbidden by the Laporte selection rules and have a low oscillator strength, and, therefore, a low intensity. The UV region of the reflectance spectra is dominated by ligand (phen) and matrix (SiO_2_) absorption bands. The 1,10-phenanthroline molecule has a strong and broad absorption band around 350 nm, which is seen as a shoulder in the reflectance spectra. This is the most important excitation channel in these complexes and the composite materials doped with them. The energy is transferred through the following path: first, the UV photon (350 nm) is absorbed by the 1,10-phenanthroline molecule and it is excited to the first singlet state. Afterward, the 1,10-phenanthroline molecule relaxes non-radiatively to its first triplet state. There onwards, through the process of intersystem crossing, the ligand molecule transfers the energy to the emitting level of the lanthanide ion. A simplified energy-transfer scheme is S_0_→S_1_→T_1_→^5^D_J_(Ln^3+^) [3,18]. Furthermore, very intense ligand-to-metal charge transfer transitions (CTTs) are visible in the UV region at around 220 nm and 260 nm. It is well-known that an O^2−^→Ln^3+^ transition is observed in oxide matrices [6,19,20]. These transitions partially overlap with some 1,10-phenanthroline peaks in the UV region (232 and 265 nm).

The excitation spectra in this work clearly show three distinct energy-transfer pathways in the luminescent [Ln(phen)_2_](NO_3_)_3_ complexes, depending on the dopant type and doping level. The first is the ligand-to-metal O^2−^→Ln^3+^ CTT at 260 nm. The second and the most intense is the ligand-to-metal resonant energy transfer described above. This transition occurs when the ligand is excited with UV light with a wavelength of around 350 nm. Similar to our previous findings, this energy transfer from 1,10-phenanthroline molecules is more efficient for the Eu^3+^ complex rather than for the Tb^3+^ one [3]. The resonant energy transfer in hybrid molecules is the most efficient when the triplet T_1_ state of the ligand is 2000–3000 cm^−1^ above the metal receiving level (^5^D_J_, J = 0, 1, 2 for Eu^3+^ and J = 3, 4 for Tb^3+^) [6]. This ensures there is little-to-no back energy transfer from the metal ion to the ligand molecule. The last energy-transfer path is via the direct excitation of the lanthanide ion (380–390 nm). This is the least-efficient excitation path due to the forbidden nature of the f-f transition. The intensity of this transition is about 1/10th of the intensity of the FRET transition visible at about 350 nm.

Luminescent materials doped with Tb^3+^ or Eu^3+^ ions are well known for their excellent light-emission properties in the green or red spectral region, which are responsible for many applications such as tricolor luminescent lamps, LED components, and sensors [19].

The emission spectra, shown in Figure 2b, display the well-known f-f transition peaks for the Eu^3+^ and Tb^3+^ emission. The mixed samples, containing Eu^3+^ and Tb^3+^, do not show any detectable emission originating from Tb^3+^ ions. This effect is discussed in the following paragraphs. Samples Eu_1, EuTb_11 and EuTb_15 have only Eu^3+^ emission peaks. We can observe ^5^D_0_-^7^F_J_ (J = 1, 2, 3) transitions. The most intense is the f-f electric dipole transition ^5^D_0_-^7^F_2_ at 615 nm, which gives the distinct red emission color of the Eu^3+^-doped composites. The only sample exhibiting green Tb^3+^ emission is the one doped with the pure [Tb(phen)_2_](NO_3_)_3_ complex, namely sample Tb_1. In Figure 2b, one can observe the ^5^D_4_-^7^F_J_ (J = 6, 5, 4, 3, 2) transitions. The most intense Tb^3+^ peak is the ^5^D_4_-^7^F_5_ transition, centered at 542 nm, giving the green color of the emission.

The optical properties of the aerogel nanocomposites are summarized in Table 1. The relative spectral intensities between the electro-dipole and magnetic-dipole transitions clearly indicate a low site symmetry, of C_2V_ or lower, around the Ln^3+^ ions [19,21]. For the [Eu(phen)_2_](NO_3_)_3_ complex, the ratio between the electro-dipole transition ^5^D_0_-^7^F_2_ and the magnetic-dipole transition ^7^D_0_-^7^F_1_ is calculated. The calculated ratios for samples Eu_1, EuTb_11 and EuTb_15 are 5.98, 5.92 and 6.06, respectively; they are lower than the calculated ratio for the pure [Eu(phen)_2_](NO_3_)_3_ complex. This indicates some structural changes and inhomogeneity forming during the in-situ formation of the nanocrystallites inside the silica gel pores. The intensity ratios for the composites are similar in value but they tend to increase when a higher concentration of Tb^3+^ is used. This indicates a lowering of the site symmetry around Eu^3+^ ions or an increased polarizability of the phen ligand in the different environments [19].

The Tb^3+^ ion’s f-f electron transitions are less site-sensitive than the electro-dipole transition ^5^D_0_-^7^F_2_ in the Eu^3+^ ion. For this reason, Tb^3+^ is rarely used as a spectroscopic probe. Nevertheless, different relative intensities in the emission peaks of the Tb^3+^ ion still indicate some structural and site changes around the ion. There is no definitive procedure to determine the site symmetry around the Tb^3+^ ion from the emission spectra, although there is one developed for Eu^3+^. The intensity ratio ^5^D_4_-^7^F_6_ to ^5^D_4_-^7^F_5_ is calculated for sample Tb_1: its value is 0.39 and is close to the ratio for the pure complex [Tb(phen)_2_](NO_3_)_3_—37 [6,22].

In Table 2, quantum yield, spectral ratio and CIE-1931 color coordinates are compared to that of the pure solid complexes. It is visible that the Tb^3+^ green f-f emission is suppressed by the Eu^3+^ doping, which indicates a possible energy transfer of 1,10-phenanthroline →Tb^3+^→Eu^3+^. Such an assumption correlates with the Diecke diagrams of the terbium and europium ions. The energy of the emitting Tb^3+^ level ^5^D_4_ (20,660 cm^−1^) is slightly above the two lowest energy levels of Eu^3+^ ion (^5^D_0_ and ^5^D_1_ with energy 17,240 and 21,550 cm^−1^, respectively). The next energy level of Tb^3+ 5^D_3_ (26,315 cm^−1^) has a similar energy to Eu^3+ 5^G_8_ and is above ^5^L_6_ (25,450 cm^−1^) and ^5^D_3_ (24,100 cm^−1^) [23,24,25]. Binnenmans et al. [6,19] predict such behavior. The relative spectral intensities are expressed through the ratio I_ED_/I_MD_, which is a semi-quantitative indicator for the site symmetry of the lanthanide ions [20]. The assumption of a Tb^3+^→Eu^3+^ transfer is supported by the relative peak intensities in the excitation spectra (Figure 2a) at 350 nm.

According to the concept of Foerster resonant energy transfer (FRET), the intensity of the transfer is inversely proportional to the distance between the species. The mean distance between the europium and terbium ions, incorporated into the pores of the aerogels, is less than 10 nm. Such an assumption is well supported by texture investigations of the silica matrix, showing a mean pore diameter of about 7 nm [4]. The in-situ formation of a binary [Eu-Tb(phen)_2_](NO_3_)_3_ nanostructure in the pore system during the two-step activation requires additional structure investigations, which will be a subject of the next contribution.

The quantum yield (QY) of the samples containing Eu^3+^ is similar to that of the pure powder complex [Eu(phen)_2_](NO_3_)_3_. The QY of the samples with europium is 30–35%, and that of the pure complex is 35%. Sample Tb_1 has a QY of 6.3%, which is half the QY of the pure Tb^3+^ complex (13%). This could be due to concentration quenching or strong non-radiative relaxation. Glasses and ceramics doped with europium usually display significant concentration quenching above 5% dopant [26]. Both the intensity ratio and CIE-1931 color coordinates suggest structural changes in the optical active species, connected with the binary doping.

### 3.2. Structure and Thermal Conductivity

It is shown that hydrophobization with trimethylchlorosilane (TMCS) leads to a decrease in thermal conductivity by about 20%, which is not dependent on the kind of dopant. It is also known that hydrophobization of the silica matrix also leads to an increase in the mean pore diameter [4]. The values of the thermal conductivity and effusivity obtained are in good agreement with the thermal conductivity vs. density dependence of porous silicate materials [16,27]. Since the thermal conductivity and effusivity of the pure crystalline dopants are very close to that of the matrix and do not affect significantly the composite properties, it can be concluded that the thermal properties of the composites are limited by the degree of hydrophobization and the pore architecture. The doping level also does not affect significantly the thermal properties of the samples.

The polymorphism observed in the present study (Figure 3) and the small size of the crystallites of [Eu(phen)_2_](NO_3_)_3_ and [Tb(phen)_2_](NO_3_)_3_ formed in the pores of the silica matrix can be explained by the strong influence of the confined spaces on the crystallization process [28]. It is known that crystallization in mesopores can lead to the formation of crystals with deformed crystal lattices that are not observed in the bulk, even leading to new crystalline phases. Crystallization in porous media can affect a number of factors, including the nucleation rate, melting and solidification temperatures, and the polymorphism, size and morphology of the formed crystals. These structural changes may be due to both kinetic and thermodynamic reasons, where it is important to note that for a given volume, the geometry of the confining medium and the interfacial energy between crystals and the confining medium will ultimately determine the effect on crystallization. The chemical composition of the pore surface, undoubtedly, plays a role in the choice of polymorphic form during crystallization from solution. A number of kinetic effects may also contribute to confined-space polymorphism. Reduced diffusion rates within small pores can result in a slow conversion between polymorphs and thus the stabilization of metastable phases [28].

### 3.3. Preparation—Properties Relation

The preparation of optical aerogel composites in this study is based on a two-step sol-gel process. In the first stage, we observe the formation of an inorganic silica aerogel matrix with a selected degree of hydrophobicity. Here, the aerogel matrix properties (hydrophobicity and pore architecture) can be tuned in order to obtain a suitable carrier for optically active species [2,3,4].

The next step is matrix functionalization. The matrix is soaked in a solution of an appropriate concentration of Ln(NO_3_)_3_ (Ln = Eu, Tb) in ethanol, followed by the addition of an ethanolic solution of 1,10-phenanthroline. After that, the granules are washed thoroughly with ethanol and dried at subcritical conditions; here, the efficacy of the drying procedure is checked by the very low thermal conductivity of the samples.

Our X-ray diffraction studies, quantum yield measurements and spectral intensity ratios demonstrate that the Tb^3+^–Eu^3+^ distance in the pores is very close (10 nm or less), resulting from the sol homogenization during the sol-gel process. There are indications of structural changes and [Eu,Tb(phen)_2_](NO_3_)_3_ nanophase formation during the functionalization of the doped-aerogel porous matrix with 1,10-phenanthroline, proven by color coordinates and luminescence spectra analysis.

A comparison between aerogel composites and sol-gel composites shows some differences [29,30]. The high porosity of the aerogel-like materials allows an increased level of doping to be achievable, compared to other sol-gel samples. The hydrophobization of the silicate matrix also increases the thermal stability of aerogel composites and lowers their thermal conductivity [3]. Compared to single-doped composites, here, the doubly doped specimens have a different excitation spectrum and show small spectral differences due to physicochemical interaction in the aerogel pores.

## 4. Conclusions

An effective two-step sol-gel approach for the preparation of aerogel-like powders, binary-doped with [Tb(phen)_2_](NO_3_)_3_ and [Eu(phen)_2_](NO_3_)_3_ nanocrystals, is demonstrated. The as-prepared hydrophobic nanocomposites have a density of about 0.2 g/cm^−3^ and a thermal conductivity of about 0.05 W/m.K, dominated by the hydrophobicity and porosity of the aerogels. Luminescence, excitation spectra and quantum yield determination strongly suggest an effective energy transfer from the 1,10-phenanthroline molecule to the Eu^3+^ ion, resulting in a quantum yield of about 30% of the red europium f-f emission. Moreover, an energy transfer from the Tb^3+^ to the Eu^3+^ ion, leading to a suppression of green terbium f-f luminescence, is observed. An indication of a lowering of the site symmetry around Eu^3+^ ions, or the increased polarizability of the 1,10-phenanthroline ligand in the presence of Tb co-doping, is evident. There is also evidence for structural polymorphism in the binary composites, proven by X-ray diffraction and luminescence spectra analysis.

## 5. Materials and Methods

### 5.1. Sol-Gel Preparation

The following chemicals were used for the syntheses of the composite materials: absolute ethanol 99.6% (abs EtOH) (Merck, Darmstadt, Germany), 65% nitric acid (Fluka, Charlotte, North Carolina, US), europium (III) oxide 99.9% (Eu_2_O_3_) (Merck, Darmstadt, Germany), terbium (III, IV) oxide 99.9% (Tb_4_O_7_) (Merck, Darmstadt, Germany) and anhydrous 1,10-phenanthroline (Merck, Darmstadt, Germany) were used without further purification.

Non-doped aerogel-like samples without hydrophobization (GR0) were prepared using a standard procedure for sol-gel oxide powders, given in [4]. The starting reagent was TEOS, followed by H_2_O addition, acid hydrolysis catalysis and basic gelation catalysis. The ratio n_TEOS_/n_H2O_ was 1:1, while the acid and basic catalyst added were n_CH3COOH_/n_TEOS_ = 1.17 and n_NH3_/n_TEOS_ = 0.04, respectively. The drying conditions applied were: room temperature and a pressure of 100 mbar.

Aerogel-like granules with a given degree of hydrophobicity α = 1.1 were synthesized through hydrolysis, solvent exchange, hydrophobization and drying using Tetraethyl orthosilicate (TEOS). Such aerogel powders have a bulk density of 0.2 g/cm^3^, a specific surface area of 870 m^2^/g and a mean pore diameter of 7 nm. Trimethyl chlorosilane (TMCS) was used as a hydrophobization agent. The degree of hydrophobicity, α, is defined as the ratio between the moles of TMCS and TEOS. More details about the subcritical process used here can be found in [4].

Solutions of Eu(NO_3_)_3_ and Tb(NO_3_)_3_ were prepared by dissolving appropriate amounts of Eu_2_O_3_ or Tb_4_O_7_ in concentrated nitric acid. After the oxides dissolved, the solutions were heated to evaporate the leftover water, and nitric acid and transparent crystals were formed. The prepared salts were dissolved in absolute EtOH to obtain solutions with a concentration of 60 mM.

For the preparation of the aerogel-like composites, a two-step colloidal route was used, starting with hydrophobic silica micropowders, as described above. In the first step, hydrophobic SiO_2_ granules were soaked in Eu(NO_3_)_3_, Tb(NO_3_)_3_ or a mixture of both. The suspension was stirred for an hour to allow the solution to fill the pores of the gel. The molar ratio of Ln/SiO_2_ (Ln = Eu, Tb) was fixed here at 0.01. In this way, both single functionalized composites (SiO_2_:0.01[Tb(phen)_2_](NO_3_)_3_ and SiO_2_:0.01[Eu(phen)_2_](NO_3_)_3_) and binary-doped composites (SiO_2_:[Eu_x_Tb_1−x_(phen)_2_](NO_3_)_3_) were obtained. For the preparation of binary-doped composites, an ethanol solution containing Eu^3+^ and Tb^3+^ nitrates was used. Afterwards, an excess solution of 1,10-phenanthroline (0.11 M is ethanol) was added, and the suspension was stirred for an additional hour. Then, the granules were filtered and washed thoroughly with ethanol, in order to remove the unreacted phen.

All samples were dried in an NÜVE vacuum oven at room temperature at a constant pressure of 100 mbar.

### 5.2. Optical Measurements

UV/Vis/NIR powder reflectance spectra were measured on an Agilent (Santa Clara, CA, USA) Cary 5000 spectrophotometer with a “Praying mantis” sample holder, using Spectralon^®^ as a reference white standard between 200 nm and 2500 nm. The Kubelka–Munk function F(R) was calculated from the diffuse reflectance of all samples. As a standard for all diffuse reflection measurements, Ho_2_O_3_ powder was used, and all peaks and relative intensities of the Ho(III) f-f transitions at 300–2000 nm were in agreement with spectral data published in [31].

The luminescence spectra of the powders were recorded on a PE (Shelton, CT, USA) FL 8500 fluorimeter equipped with an integrating sphere (N4201017) for absolute quantum yield (QY) measurements. The samples were placed in a quartz cell for the QY measurements. The absolute QY was obtained using the calculation method of Suzuki et al. [32]. The error of the QY determination here was about 5%.

Emission and excitation spectra were recorded using a variable-angle solid sample holder (N4201014) equipped with a precision cell for powder samples (N4201032). The calculation of x and y values was performed using a home-made program able to read the raw data file as it comes out from the instrument, based on the procedure published in [16]. The data points were validated through the OSRAM Sylvanya Color Calculator program. All the diffuse reflectance, excitation and emission spectra were quantified using Gaussian deconvolution.

### 5.3. X-ray Diffraction and Thermal Properties

The structure and microstructure of the samples were characterized by X-ray diffraction with Cu-Kα radiation (Panalytical (Almelo, Netherlands) Empyrean 3 diffractometer). The mean crystallite sizes were calculated using Scherrer’s equation for the most intense peak of [Ln(phen)_2_](NO_3_)_3_. The error of the lattice constant determination was about 0.005 Å. A qualitative phase analysis was performed on the following structures: [Tb(phen)_2_](NO_3_)_3_, [Eu(phen)_2_](NO_3_)_3_, 1,10-phenanthroline, Tb(NO_3_)_3_, and Eu(NO_3_)_3_.

The thermal conductivities were measured on a C-Therm (C-Therm Technologies Ltd., Fredericton, NB, Canada) MTPS (Modified Transient Plane Source) TCi Thermal Conductivity Analyzer equipped with a powder and liquid sample holder. The error of the thermal conductivity determination of the gel powders was about 0.0002 W/mּ.K. At these conditions, the thermal properties of hydrophobic Cabot Lumira aerogel granules, λ = 0.0025 W/m.K and e = 29.8 W.s ^1/2^/m^2^.K at 15.6 °C, were obtained as an external reference.

### 5.4. Color Coordinates

Converting spectra to RGB (Red, Green, Blue) coordinates is a relatively common procedure in optical spectroscopy and analysis. The conversion allows for the visualization of fluorescence data in a color format, which is easily interpretable by the human eye. The acquired spectra were normalized and the converted to XYZ coordinates by multiplying the spectral data by the respective color-matching curves [33]. The XYZ color coordinates were transferred to RGB (Red, Green, Blue) using the standard matrix conversion, with a reference white point of E = (0.333, 0.333, 0.333), an sRGB color space, and Gamma correction (1.0) [34]. The values obtained in this procedure were compared with those obtained from free programs and websites, and full agreement was found.

## Figures and Tables

**Table 1:** Thermal properties of the investigated samples at room temperature. The experimental error of the density is about 5%.

Sample	α	λ W/m.K	Δλ W/m.K	e W.s^1/2^/m^2^.K	Δe W.s^1/2^/m^2^.K	ρ g/cm^3^	n_Ln_/n_SiO2_
GR0	0	0.0640	0.0002	145.3896	0.8531	0.45	0
Eu_1	1.1	0.0523	0.0001	95.6041	0.5516	0.23	0.01
Tb_1	1.1	0.0467	0.0001	71.2591	0.5505	0.18	0.01
EuTb_11	1.1	0.0510	0.0001	90.2086	0.4896	0.18	0.01
EuTb_15	1.1	0.0523	0.0003	95.5750	1.1232	0.18	0.01

**Table 2:** Summary of the optical properties of the composite samples: sample name, chemical composition, quantum yield, relative intensity ratio and CIE-1931 color coordinates of the emission.

Sample	Chemistry	QY, Red %	QY, Green %	I_ED_/I_MD_	x	y	z
GR0	SiO_2_	-	-	-	-	-	-
Tb_1	SiO_2_:0.01Tb	-	6.3	0.39	0.631	0.340	0.03
Eu_1	SiO_2_:0.01Eu	35	-	5.98	0.358	0.486	0.156
EuTb1_1	SiO_2_:0.005Eu;0.005Tb	30	-	5.92	0.626	0.336	0.038
EuTb1_5	SiO_2_:0.0017Eu;0.0083Tb	32	-	6.06	0.597	0.322	0.081
Euphen	[Eu(phen)_2_](NO_3_)_3_ [2,3]	35	-	7.25	0.309	0.602	0.089
Tbphen	[Tb(phen)_2_](NO_3_)_3_ [2,3]	-	13	0.37	0.665	0.333	0.003

## Data Availability

The data presented in this study are openly available in article.
